# Supplementary material for: Visualizing suborganellar lipid distribution using correlative light and electron microscopy
Source: Nat Cell Biol. 2026 Mar 20;28(4):828–37. doi: 10.1038/s41556-026-01915-x (PMC13086582; doi:10.1038/s41556-026-01915-x)
Supplement: Supplementary file 1 — Supplementary Figs. 1–9. [file 41556_2026_1915_MOESM1_ESM.pdf]

nature cell biology

Supplementary information

https://doi.org/10.1038/s41556-026-01915-x

Visualizing suborganellar lipid distribution using correlative light and electron microscopy

In the format provided by the authors and unedited

Supplementary Information

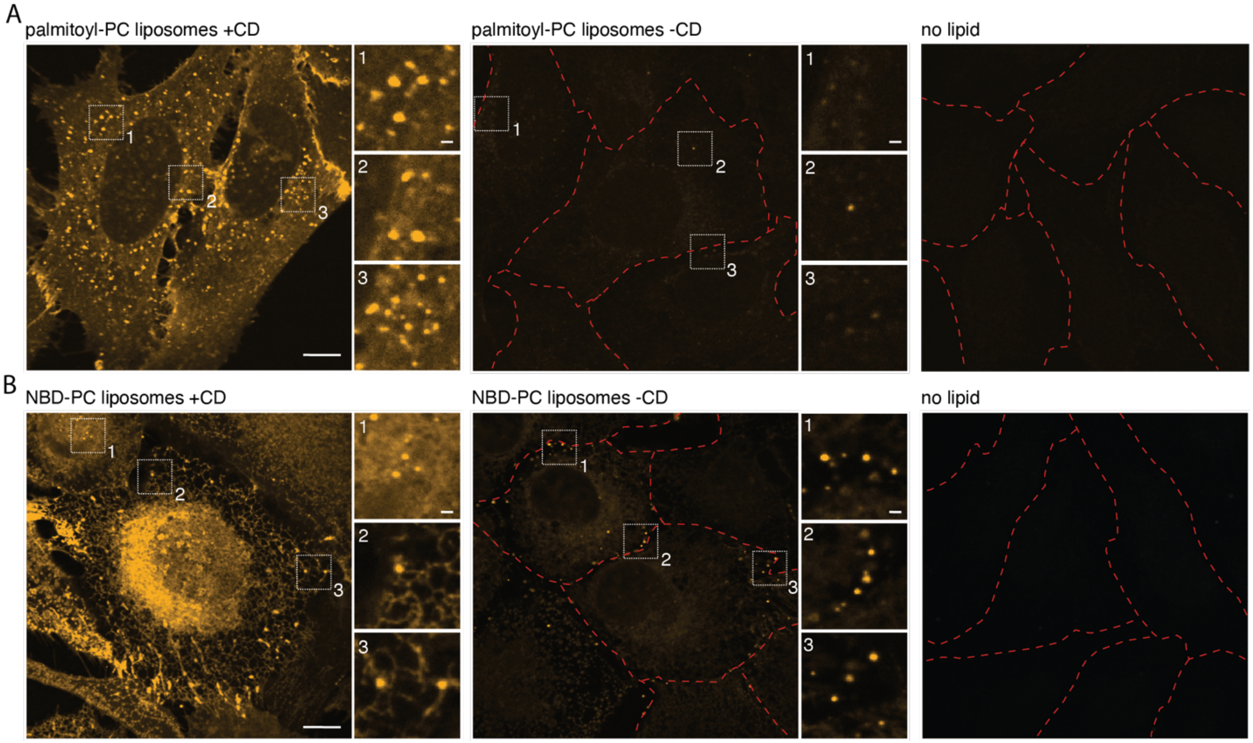

Supplementary Figure 1| Bifunctional lipid probe-loaded liposomes are not taken up during lipid feeding. **A**: U2OS wildtype cells were treated with bifunctional lipid containing liposomes preincubated with alpha methyl cyclodextrin (+CD) or not (-CD). As a control samples without lipid treatment were also stained (no lipid). The samples without CD showed a low background, suggesting no uptake of whole liposomes by endocytosis. **B**: To ensure that the triton wash did not falsify this result, U2OS wildtype cells were also incubated with a mix of NBD-PC containing liposomes with and without CD during live cell imaging. Similarly low background was detected for loading conditions without CD. Scale bars: 10 µm/ 1 µm. The experiment was performed for one biological replicate.

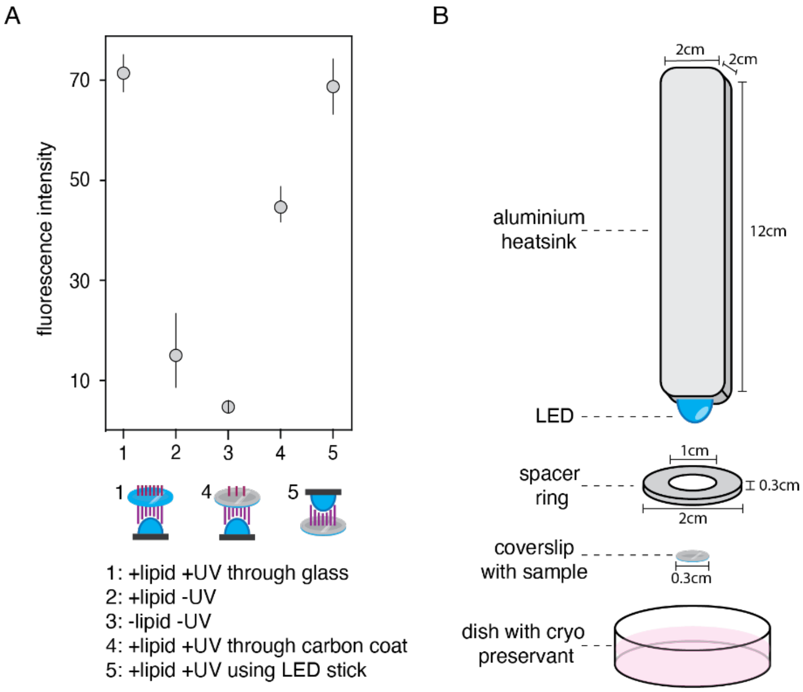

Supplementary Figure 2| A water-resistant UV-LED setup allows for effective photo-crosslinking of carbon-coated samples. **A**: UV crosslinking efficiencies were measured in U2OS cells loaded with PC(16:0|Y). UV crosslinking was performed through glass (1), carbon coated glass (4) or from above (5). Negative controls were not treated with UV light (2) or PC(16:0|Y) (3). Mean values of fluorescent images are shown, error bars represent the standard deviation. The experiment was performed for 3 technical replicates (n(field of view): 27). **B**: Schematic representation of the LED setup for illuminating samples in medium. Samples on coverslips are placed in a dish, a spacer ring is placed between the sample and the LED stick.

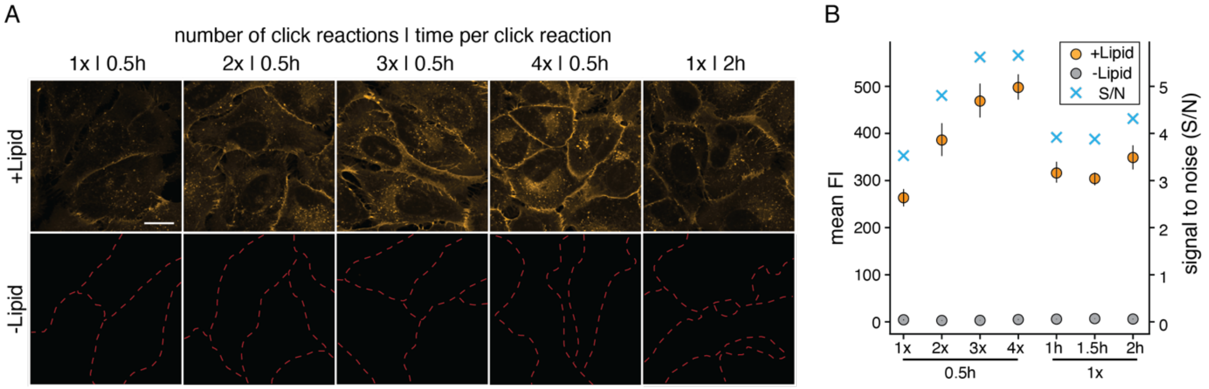

Supplementary Figure 3| Repeated click labeling increases fluorescence signal. A: Representative fluorescence images of the optimization of the copper catalyzed click reaction are shown. U2OS wildtype cells were loaded for 4 min with PC(16:0|Y). Samples were stained 1 – 4 times for 30 min each, or stained once for the corresponding prolonged time. Negative controls (-Lipid) are shown in the bottom panels. Dotted red lines show the cellular outlines if not visible otherwise. Scale bar: 20 µm. **B**: Quantification of the experiment. Background corrected mean values of 3 technical replicates (n(field of view): 27) are shown. Error bars are shown as standard deviation. Lipid loaded samples are indicated in orange, negative controls in grey and the signal to noise ratio (S/N) as blue crosses.

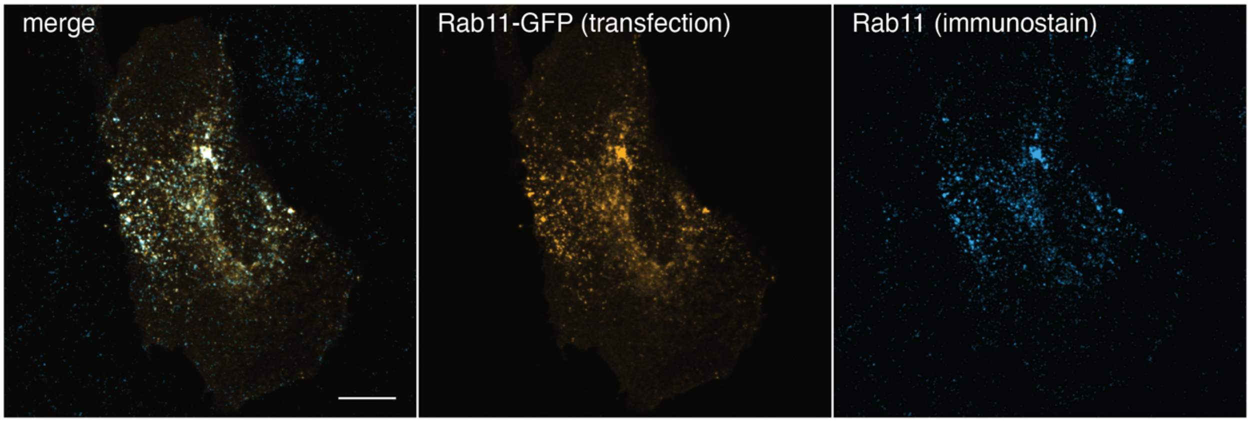

Supplementary Figure 4| A Rab11 antibody binds Rab11 specifically. U2OS wildtype cells were transfected to express Rab11-GFP (orange). Samples were fixed and immunostained against Rab11 using a Rab11 antibody (blue). Scale bar: 10 µm. The experiment was performed for one biological replicate.

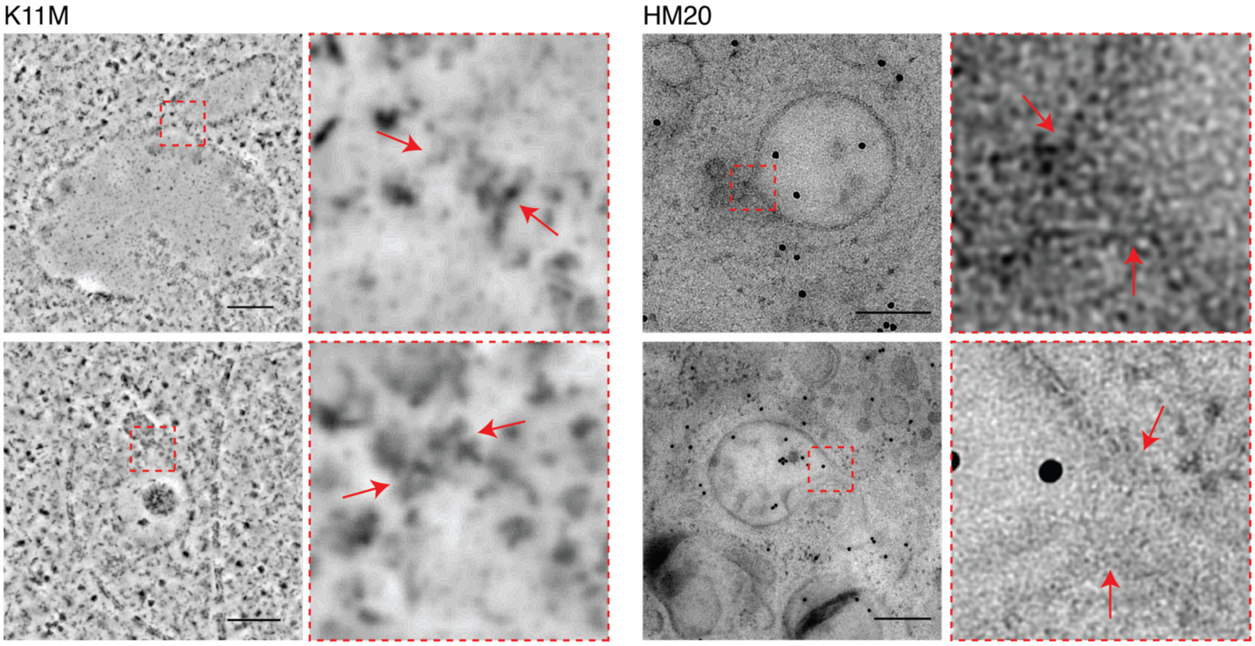

Supplementary Figure 5| Connectivity of recycling tubules to early endosomes. Exemplary early endosomes embedded in K11M and HM20 are shown. The area where the boundary membrane transitions to recycling tubules is highlighted and shown magnified. Red arrows highlight the neck region. Scale bars: 200 nm.

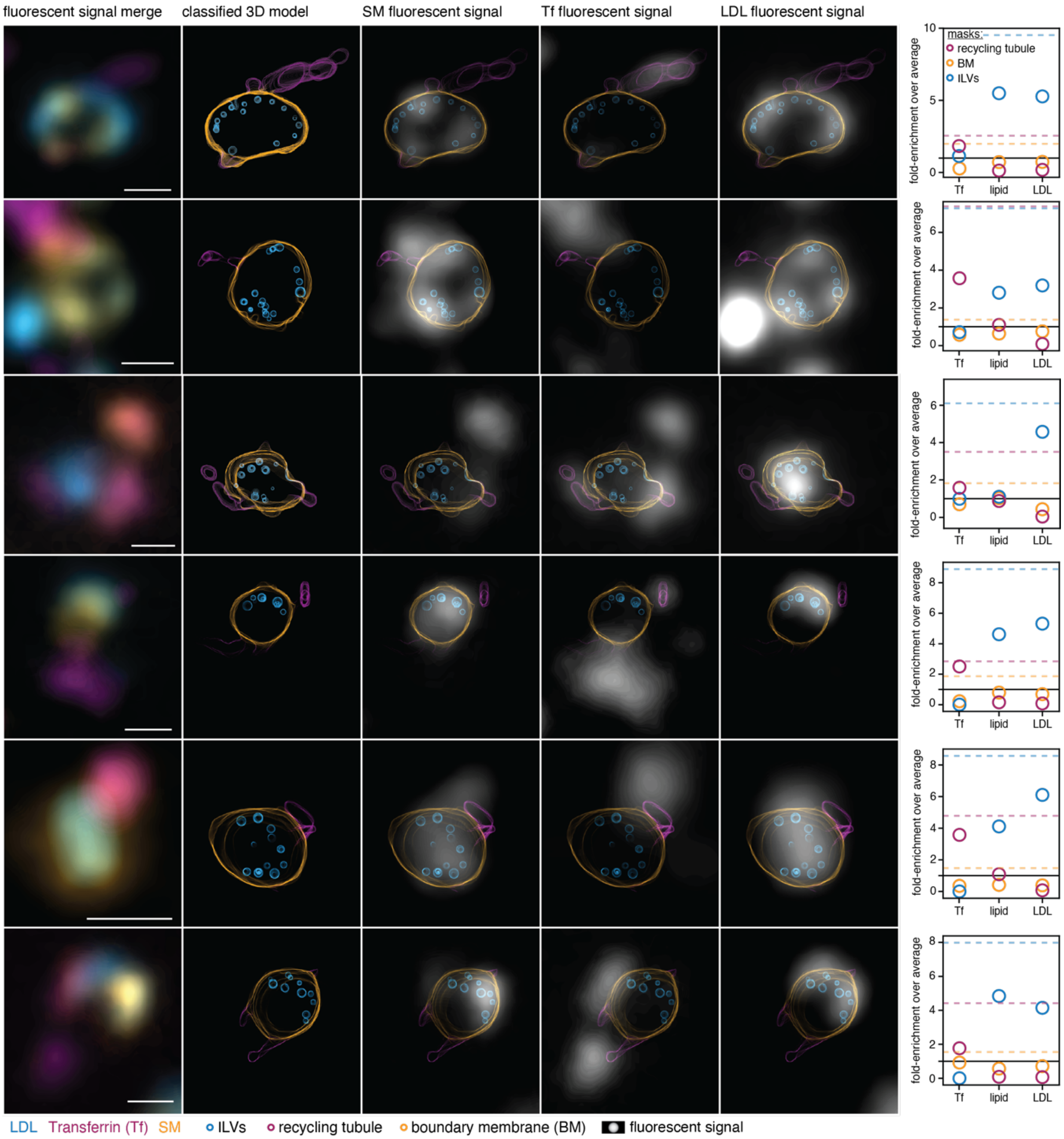

Supplementary Figure 6| Analysis of early endosomes loaded with bifunctional sphingomyelin. All endosomes analyzed for the first biological replicate loaded with sphingomyelin (SM) are shown. The endosomes of row 1 and row 3 are shown also in the main text Figure 4. Column 1 shows the overlay of LDL (blue), transferrin (magenta) and SM (orange) fluorescence signals. Column 2 shows the max projected line profile of the classified 3D model. Column 3-5 show the model overlays with the fluorescent signal of SM, transferrin or LDL from left to right, respectively. On the right most side the enrichments of signal densities are depicted for all fluorescent signals over all membrane classes. Horizontal lines mark the maximum fluorescent density for recycling tubules (magenta dotted), intraluminal vesicles (blue dotted), the boundary membrane (orange dotted) as well as no enrichment (black). Scale bars: 500 nm.

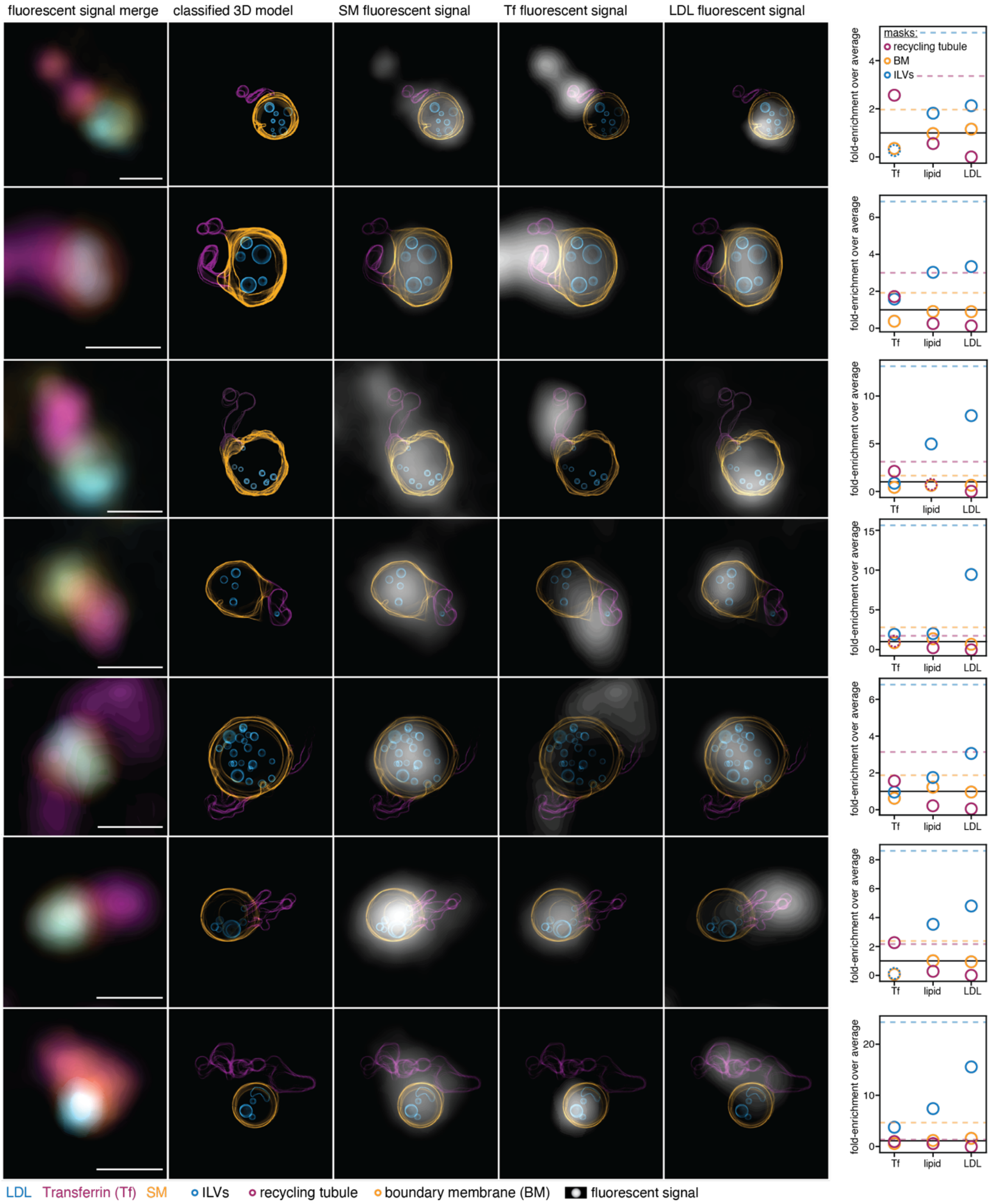

Supplementary Figure 7| Analysis of early endosomes loaded with bifunctional sphingomyelin. All endosomes analyzed for the second biological replicate loaded with sphingomyelin (SM) are shown. The endosomes of row 3 are shown also in the main text Figure 4. Column 1 shows the fluorescent overlay of LDL (blue), transferrin (magenta) and SM (orange). Column 2 shows the max projected line profile of the classified 3D model. Column 3-5 show the model overlays with the fluorescent signal of SM, transferrin or LDL from left to right, respectively. On the right most side the fold enrichments of signal densities are depicted for all fluorescent signals over all membrane classes.

Horizontal lines mark the maximum fluorescent density for recycling tubules (magenta dotted), intraluminal vesicles (blue dotted), the boundary membrane (orange dotted) as well as no enrichment (black). Scale bars: 500 nm.

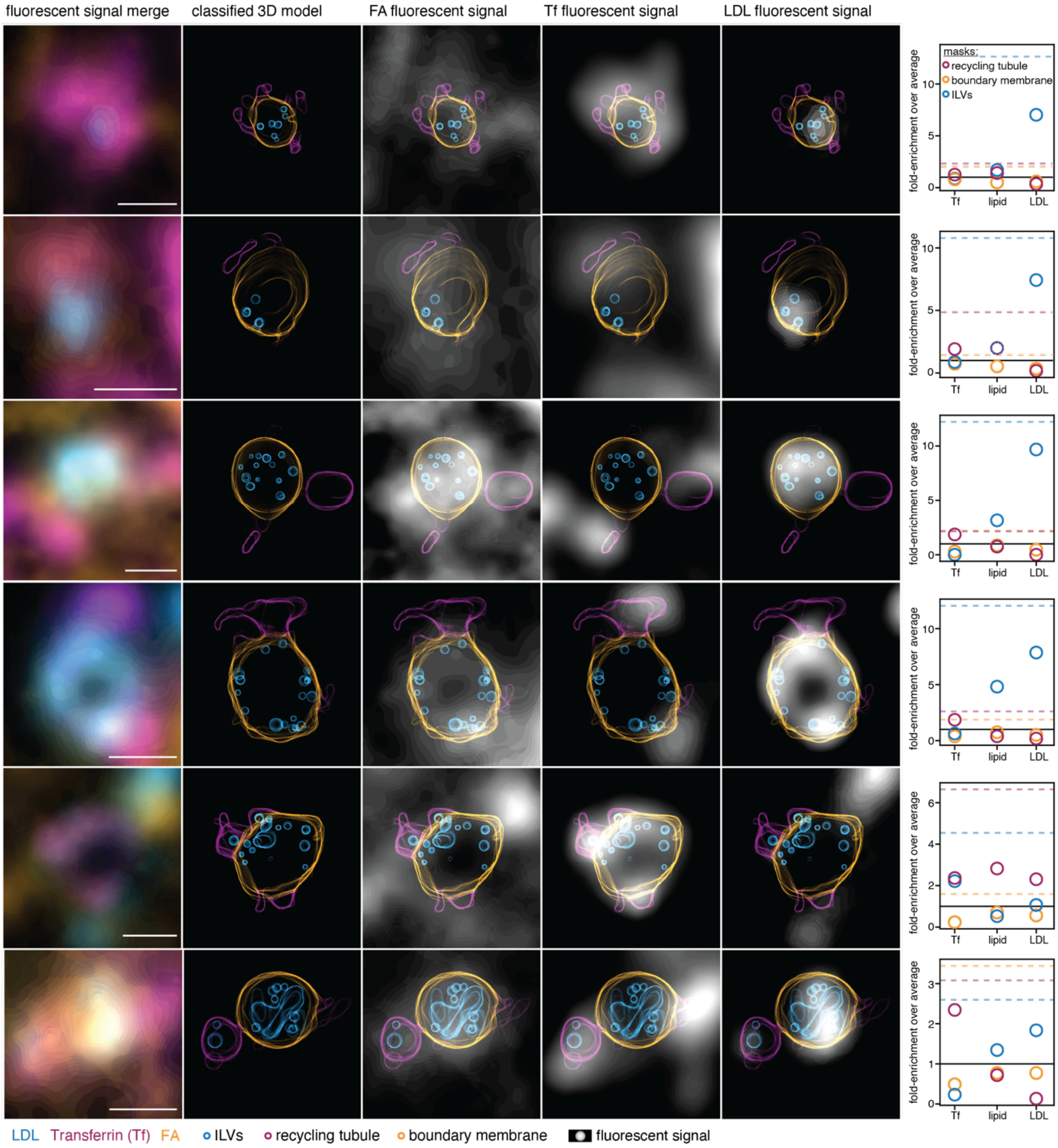

Supplementary Figure 8| Analysis of early endosomes loaded with bifunctional fatty acid. All endosomes analyzed for the first biological replicate metabolically labelled with bifunctional palmitic acid (Y). Column 1 shows the fluorescent overlay of LDL (blue), transferrin (magenta) and SM (orange). Column 2 shows the max projected line profile of the classified 3D model. Column 3-5 show the model overlays with the fluorescent signal of SM, transferrin or LDL from left to right, respectively. On the right most side the fold enrichments of signal densities are depicted for all fluorescent signals over all membrane classes. Horizontal lines mark the maximum fluorescent density for recycling tubules (magenta dotted), intraluminal vesicles (blue dotted), the boundary membrane (orange dotted) as well as no enrichment (black). Scale bars: 500 nm.

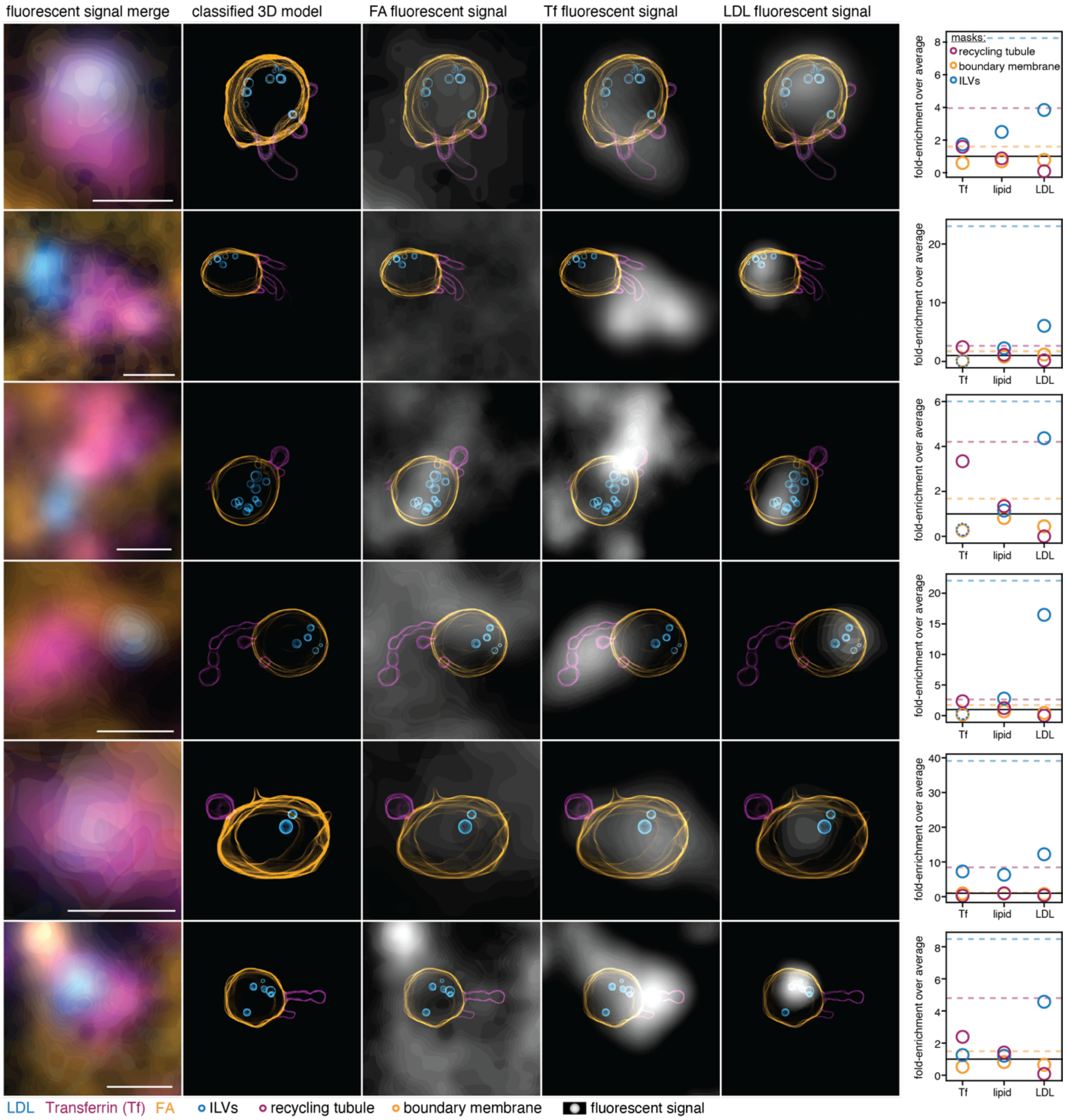

Supplementary Figure 9| Analysis of early endosomes loaded with bifunctional fatty acid. All endosomes analyzed for the second biological replicate metabolically labelled with bifunctional palmitic acid (Y). The endosome of row 1 is shown also in the main text Figure 4. Column 1 shows the fluorescent overlay of LDL (blue), transferrin (magenta) and SM (orange). Column 2 shows the max projected line profile of the classified 3D model. Column 3-5 show the model overlays with the fluorescent signal of SM, transferrin or LDL from left to right, respectively. On the right most side the fold enrichments of signal densities are depicted for all fluorescent signals over all membrane classes. Horizontal lines mark the maximum fluorescent density for recycling tubules (magenta dotted), intraluminal vesicles (blue dotted), the boundary membrane (orange dotted) as well as no enrichment (black). Scale bars: 500 nm.
